# Most-Probable-Number-Based Minimum Duration of Killing Assay for Determining the Spectrum of Rifampicin Susceptibility in Clinical Mycobacterium tuberculosis Isolates

**DOI:** 10.1128/AAC.01439-20

**Published:** 2021-02-17

**Authors:** Srinivasan Vijay, Hoang Ngoc Nhung, Nguyen Le Hoai Bao, Do Dang Anh Thu, Le Pham Tien Trieu, Nguyen Hoan Phu, Guy E. Thwaites, Babak Javid, Nguyen T. T. Thuong

**Affiliations:** a Oxford University Clinical Research Unit, Ho Chi Minh City, Vietnam; b Centre for Tropical Medicine and Global Health, Nuffield Department of Medicine, University of Oxford, Oxford, United Kingdom; c School of Medicine, Vietnam National University of Ho Chi Minh City, Ho Chi Minh City, Vietnam; d Centre for Infectious Diseases and Research, Tsinghua University School of Medicine, Beijing, China; e Beijing Advanced Innovation Center in Structural Biology, Beijing, China; f Division of Experimental Medicine, University of California, San Francisco, California, USA

**Keywords:** *Mycobacterium tuberculosis*, rifampicin, antibiotic tolerance assay, minimum duration of killing, most probable number, MDK, MPN, antibiotic tolerance

## Abstract

Accurate antibiotic susceptibility testing is essential for successful tuberculosis treatment. Recent studies have highlighted the limitations of MIC-based phenotypic susceptibility methods in detecting other aspects of antibiotic susceptibilities in bacteria. Duration and peak of antibiotic exposure, at or above the MIC required for killing the bacterial population, has emerged as another important factor for determining antibiotic susceptibility. This is broadly defined as antibiotic tolerance. Antibiotic tolerance can further facilitate the emergence of antibiotic resistance. Currently, there are limited methods to quantify antibiotic tolerance among clinical M. tuberculosis isolates. In this study, we develop a most-probable-number (MPN)-based minimum duration of killing (MDK) assay to quantify the spectrum of M. tuberculosis rifampicin susceptibility within subpopulations based on the duration of rifampicin exposure required for killing the bacterial population. MDK_90_–_99_ and MDK_99.99_ were defined as the minimum duration of antibiotic exposure at or above the MIC required for killing 90 to 99% and 99.99% of the initial (pretreatment) bacterial population, respectively. Results from the rifampicin MDK assay applied to 28 laboratory and clinical M. tuberculosis isolates showed that there is variation in rifampicin susceptibility among isolates. The rifampicin MDK_99_**_/_**_99.99_ time for isolates varied from less than 2 to 10 days. MDK was correlated with larger subpopulations of M. tuberculosis from clinical isolates that were rifampicin tolerant. Our study demonstrates the utility of MDK assays to measure the variation in antibiotic tolerance among clinical M. tuberculosis isolates and further expands clinically important aspects of antibiotic susceptibility testing.

## Abstract (precis)

Accurate antibiotic susceptibility testing is essential for successful tuberculosis treatment. Recent studies have highlighted the limitations of MIC-based phenotypic susceptibility methods in detecting other aspects of antibiotic susceptibilities in bacteria. Duration and peak of antibiotic exposure, at or above the MIC required for killing the bacterial population, has emerged as another important factor for determining antibiotic susceptibility. This is broadly defined as antibiotic tolerance.

## INTRODUCTION

Tuberculosis (TB), caused by Mycobacterium tuberculosis, results in more than 1.5 million deaths a year (1). Although TB can be successfully treated with antibiotics, a minimum of 6 months of treatment is required (2). Relapse posttreatment and emergence of antibiotic resistance are major negative sequelae of inadequate treatment (2). Success in therapy requires the accurate determination of antibiotic susceptibility and initiation and adherence to an effective antibiotic regimen (3).

The most common measure of antibiotic susceptibility of M. tuberculosis isolates is the MIC (4), the concentration of antibiotics that inhibits, kills, or reduces the growth of at least 99% of the bacterial population (5). The MICs for antibiotics vary between clinical M. tuberculosis isolates, and epidemiologically relevant MIC breakpoints are used to define M. tuberculosis isolates as susceptible or resistant to each antibiotic (6).

However, Colangeli et al. recently showed that MICs in the susceptible range, below the clinical cutoff for resistance, can be associated with poor treatment outcomes in TB (7). Furthermore, recent findings in other bacteria suggest that antibiotic susceptibility differences can even emerge between isolates with similar MICs due to differences in the duration of antibiotic exposure required for killing bacterial subpopulations (8). Therefore, susceptibility or resistance to an antibiotic, defined by the clinical MIC cutoff, is not the only measure of antimicrobial susceptibility, as bacterial isolates can still display widely different susceptibilities to bactericidal antibiotics, a phenomenon broadly described as antibiotic tolerance (8, 9). With antibiotic tolerance, subpopulations of bacteria that are genetically susceptible to an antibiotic are killed more slowly than the bulk population (8). Studies have identified the emergence of mutations in M. tuberculosis clinical isolates associated with increased levels of antibiotic tolerance (10–13). The emergence of antibiotic tolerance can further facilitate the emergence of resistance (14–17). These studies highlight limitations of reliance on clinical MIC cutoff alone as the measure of antibiotic susceptibility and the possible role of antibiotic tolerance in poor treatment progression and evolution of antibiotic resistance in M. tuberculosis isolates. Hence, it is important to investigate the level of antibiotic tolerance among clinical M. tuberculosis isolates defined as susceptible based on clinical MIC cutoffs for resistance.

One way to measure the level of antibiotic tolerance is to determine the duration of antibiotic exposure at or above MICs required for killing the majority of the bacterial population (18). Kill-curve assays allow the study of the dynamics of bacterial population killing after antibiotic exposure (19). Antibiotic-mediated killing of bacterial populations is biphasic, with an initial rapid killing of the majority population followed by a low rate of killing of minority populations, also known as tolerant and persistent bacterial populations (19). Repeated antibiotic exposure can lead to an increase in the level of antibiotic tolerance both at subpopulation and bulk population levels (11, 20). This will further increase the time required for killing the bacterial population. Recent clinical studies have shown in-host evolution of antibiotic tolerance in other pathogenic bacteria and its association with treatment complications (21).

Therefore, quantitative assays to measure the antibiotic tolerance of bacterial populations are required. Commonly used tolerance assays are based on the duration required for killing the bacterial population by antibiotics, also known as the minimum duration of killing (MDK) assay (18). The MDK assay can be used to measure both bulk population and subpopulation levels of antibiotic tolerance (18). MDK_90_ and MDK_99_ refer to the minimum duration required for killing 1 or 2 log_10_ fold (90% to 99%) or the majority of the bacterial population during antibiotic exposure. Hence, MDK_90_ or MDK_99_ can quantify the bulk population level of antibiotic tolerance (18), whereas MDK_99.99_ determines the minimum duration required for killing 4 log_10_ fold of the population and, assuming the tolerant subpopulation is >0.1%, quantifies the subpopulation level of antibiotic persistence (18).

Reliable and feasible methods are required to measure MDK values of clinical M. tuberculosis isolates to determine if there are significant variations in antibiotic tolerance among M. tuberculosis isolates. A critical measure used in the MDK assay is to accurately determine the viable bacterial numbers at different time points after antibiotic exposure of the bacterial culture. Usually, bacterial viability is measured by either CFU enumeration or most-probable-number (MPN) methods (22). In the MPN method, the viable bacterial numbers in culture are determined by generating 10-fold serial dilutions of the culture until limiting dilution (i.e., the dilutions show no visible bacterial growth over time) is reached. The viable bacterial number (MPN/ml) is calculated by considering that the highest dilution showing growth contains at least one viable bacterium and then multiplying by the dilution factor (23). Previous study has shown that MPN and CFU methods give comparable viable counts for M. tuberculosis under starvation in phosphate-buffered saline (PBS) (24), whereas only the MPN method can detect differentially detectable M. tuberculosis cells generated under rifampicin treatment after initial starvation in PBS, as such M. tuberculosis cells fail to grow in the CFU method (24). Furthermore, the MPN method facilitates differentially culturable M. tuberculosis growth from clinical samples (25), increasing the count of viable bacteria compared to the CFU method. In addition, all serial dilutions can be simultaneously incubated in one microtiter plate in the MPN method, overcoming the laborious process of plating multiple serial dilutions to obtain countable numbers of colonies using the CFU method (26). Considering these advantages of the MPN method, we applied this method for determining bacterial viability in our MDK assay.

Given the prime importance of rifampicin in drug-sensitive tuberculosis treatment (27), we developed and tested an MPN-based MDK assay for determining the level of rifampicin tolerance among M. tuberculosis isolates. We applied this assay on a pilot scale to determine the spectrum of rifampicin susceptibility in 27 clinical M. tuberculosis isolates and identified variation in rifampicin susceptibility among these isolates, which included 7 isoniazid- and rifampicin-susceptible isolates, 19 isoniazid-resistant isolates, and 1 multidrug-resistant TB (MDR-TB) isolate, resistant to both isoniazid and rifampicin.

## RESULTS

### Developing an MPN-based MDK assay for determining M. tuberculosis rifampicin susceptibility.

**FIG 1:**
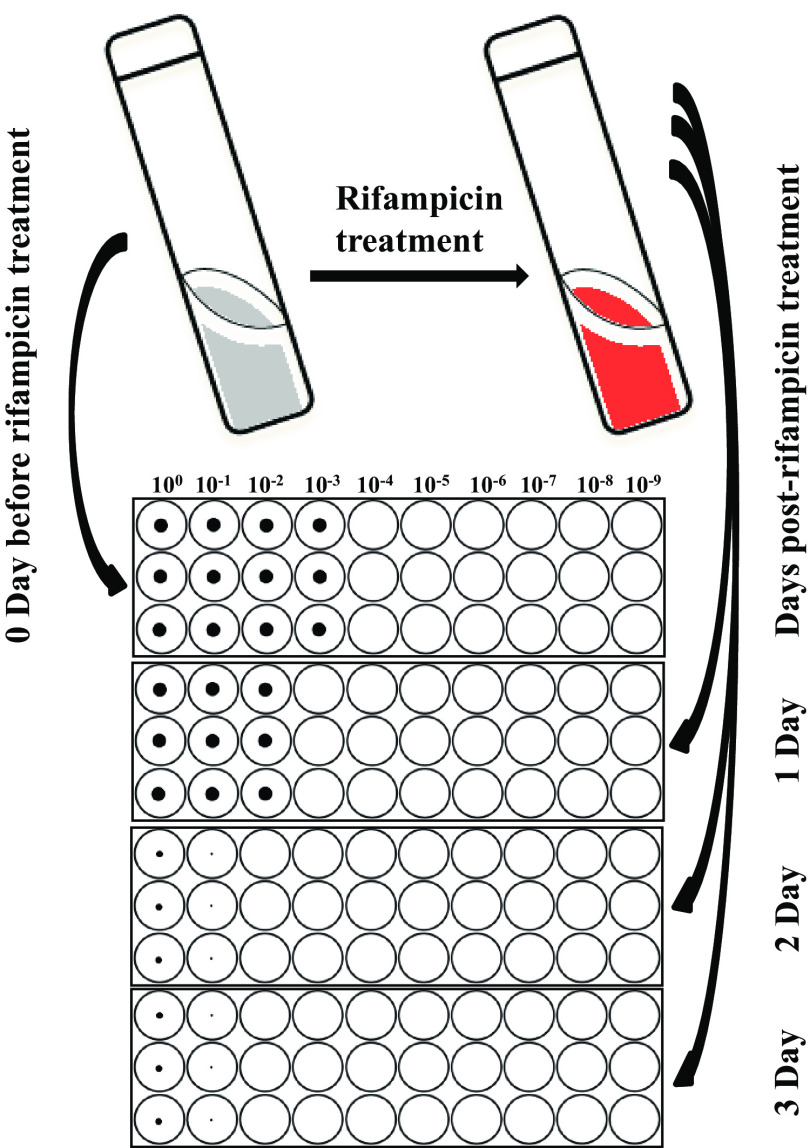
MDK assay study design. Mycobacterial cultures were grown in 50-ml tubes until the OD reached 0.4 to 0.6. This culture was diluted to an OD of 0.4 with fresh 7H9T medium. One milliliter from this culture was removed at day 0 (just before rifampicin treatment) for measuring viable bacterial numbers by serial dilution in triplicate using 96-well microtiter plates. The serially diluted microtiter plates were incubated for 1 to 2 months for determining the MPN/ml. After day 0 sampling, the remaining culture in the tube was treated with rifampicin (1 or 2 μg/ml) and incubated further. At different time points after rifampicin treatment, 1 ml rifampicin-treated culture was removed and centrifuged, and the cell pellet was washed free of antibiotic, resuspended in fresh medium, and serially diluted similarly to the day 0 procedure. The bacterial number in the original culture before and after rifampicin treatment is determined by the MPN method.

**FIG 2:**
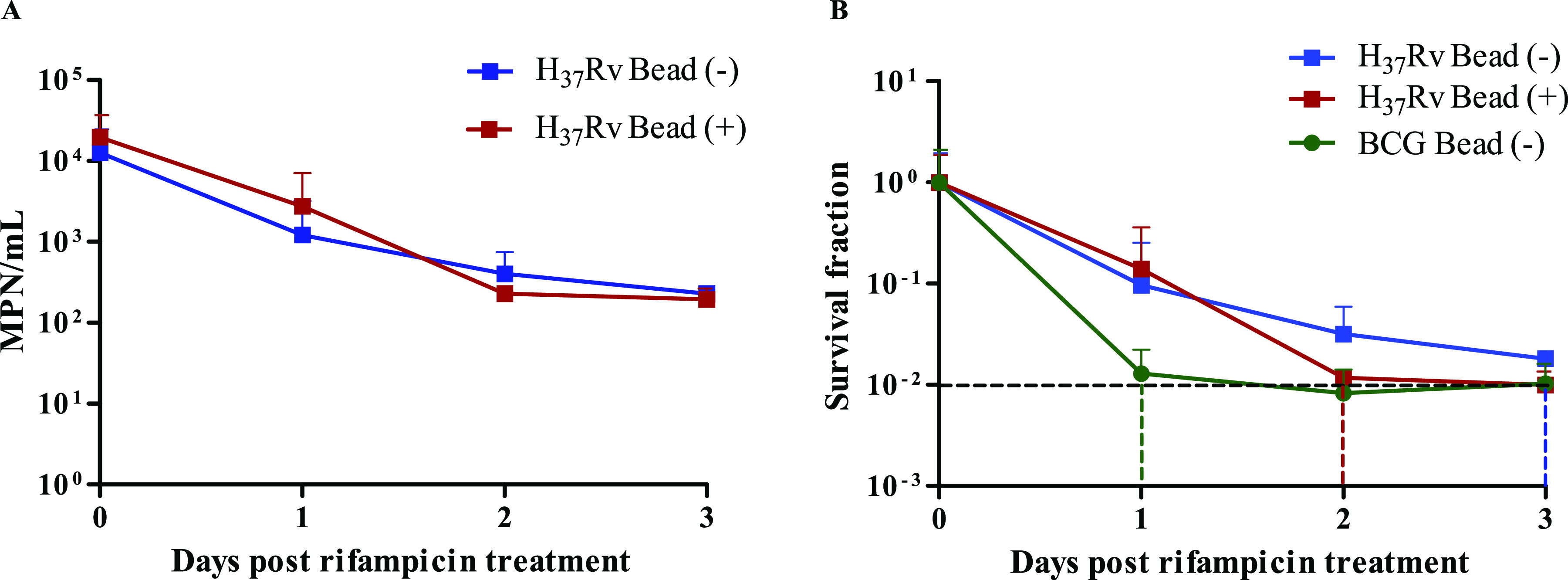
MPN-based MDK assay development using laboratory mycobacterial strains M. tuberculosis H37Rv and M. bovis BCG. (A) Mid-log-phase cultures of laboratory strain H37Rv (with [+] and without [−] beads) were treated with 1 μg/ml rifampicin, and viable bacterial numbers were measured as MPN/ml just before rifampicin treatment (day 0) and each day after rifampicin treatment for 3 days. (B) Survival fraction and MDK_99_ of M. tuberculosis H37Rv and M. bovis BCG. The black dashed horizontal line indicates a 2-log_10_ fold reduction in survival fraction (MDK_99_) compared to day 0. Dashed colored vertical lines indicate the MDK_99_ time for individual isolates with or without beads. The data are average MPN/ml at each day for 4 experiments. M. tuberculosis H37Rv experiments with and without beads are marked with different colors.

To develop the MDK assay and to determine rifampicin susceptibility, we initially optimized the assay by testing laboratory strains M. tuberculosis H37Rv and M. bovis BCG treated with rifampicin in mid-log phase (Fig. 1 and 2A and B). Both laboratory strains showed rapid killing with 1 μg/ml rifampicin treatment, the cutoff concentration used for defining susceptible and resistant isolates in mycobacterial growth indicator tube (MGIT) culture for clinical isolates (28). The decrease in MPN/ml compared with day 0 in microtiter plates showed an MDK_99_ time of less than 1 day for M. bovis BCG and between 2 and 3 days after rifampicin treatment for H37Rv with and without bead beating (Fig. 2B). Bead beating to remove bacterial aggregation, in addition to Tween 80 being in the culture media, marginally decreased H37Rv rifampicin tolerance compared to that of cultures with Tween 80 alone (without beads).

### Factors influencing rifampicin MDK assay for M. tuberculosis isolates.

Some of the factors that need to be considered for the rifampicin MDK assay are the influence of nutrients, such as carbon source, on M. tuberculosis survival and growth in the MDK assay; we determined the M. tuberculosis viable count in the presence or absence of glycerol by the MPN method (see Fig. S1A in the supplemental material). The results showed that the presence of glycerol in the culture medium consistently increased the MPN compared to those of cultures without glycerol, indicating the influence of carbon source on M. tuberculosis viable numbers.

Similarly, washing was done with 7H9T medium to minimize the changes in culture conditions for the bacterium during different steps of the MDK assay. The washing step is critical, and care should be taken to prevent bacterial cell loss during removal of supernatant. The washing must be adequate to remove rifampicin, as residual rifampicin may inhibit M. tuberculosis growth at undiluted or lower dilutions in MPN dilution series (Fig. S1C). These aspects were considered for standardizing optimum washing steps in the MDK assay.

In addition, initially we performed the rifampicin MDK assay using the CFU method for H37Rv (Fig. S2A). Although we observed similar patterns of kill curves for 20 days of rifampicin treatment between CFU and MPN methods (Fig. S2), the CFU method was difficult to apply to a large set of clinical M. tuberculosis isolates, as getting countable numbers of colonies required plating multiple serial dilutions, which became laborious. Therefore, we applied an MPN-based rifampicin MDK assay for clinical M. tuberculosis isolates.

### Applying rifampicin MDK assay for clinical M. tuberculosis isolates.

**FIG 3:**
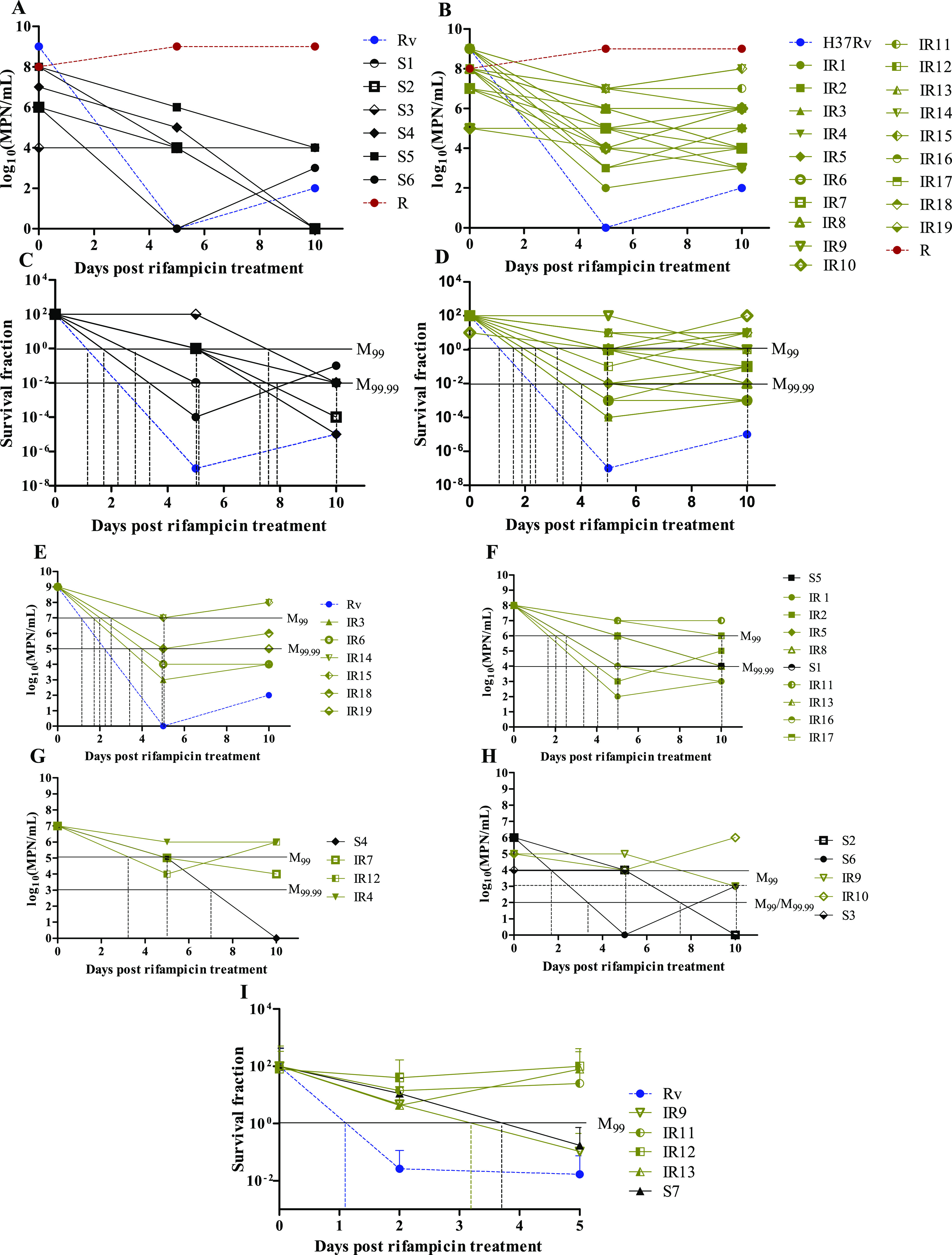
Applying rifampicin MDK assay to clinical M. tuberculosis isolates. (A and B) rifampicin MDK assay for mid-log-phase cultures of 6 clinical M. tuberculosis isolates susceptible to isoniazid and rifampicin (S1 to S6) (A), and 19 isoniazid-resistant isolates (IR1 to IR19) were treated with 2 μg/ml rifampicin (B). Viable mycobacterial cell numbers were determined by the MPN method on day 0 (just before rifampicin treatment) and 5 and 10 days after rifampicin treatment. Laboratory strain H37Rv (Rv) and MDR-TB (R) isolates were used as controls for low and high rifampicin tolerance, respectively, with both susceptible and isoniazid-resistant isolates. (C and D) Normalized survival fraction of susceptible (C) and isoniazid-resistant (D) isolates to determine the MDK_99_ and MDK_99.99_ times. Black solid horizontal lines indicate 2-log_10_ fold (M_99_) and 4-log_10_ fold (M_99.99_) reductions in survival fractions compared to day 0 values (normalized as 100% for susceptible and isoniazid-resistant isolates). Black dashed vertical lines show the approximate time required for 99% or 99.99% reduction in survival fraction of individual M. tuberculosis isolates. (E to G) Susceptible and isoniazid-resistant isolates (from panels A and B) grouped based on decreasing order of their initial MPN counts. (H) M. tuberculosis isolates with initial log_10_ MPN of 6, 5, and 4 grouped together, as there were only a few isolates in each of these groups for MDK time determination. (I) Normalized survival fraction of the H37Rv subset of isoniazid-resistant (IR9, IR11, IR12, and IR13) and a susceptible isolate (S7) at 0, 2, and 5 days after rifampicin treatment to determine the MDK_99_ duration (3 to 6 biologically independent experiments). Black solid horizontal lines indicate 2-log_10_ fold (M_99_) reduction in survival fraction compared to day 0 values (normalized as 100% for all M. tuberculosis strains). Dashed vertical lines show approximate times required for 99% reduction in survival fraction of individual M. tuberculosis isolates, except for three isoniazid-resistant isolates with high rifampicin tolerance (IR11, IR12, and IR13, M_99_ of >5 days).

We then applied the MDK assay to investigate variation in rifampicin susceptibility among clinical M. tuberculosis isolates (*n* = 27). Clinical M. tuberculosis isolates were selected based on GeneXpert MTB/RIF results for rifampicin susceptibility and phenotypic drug susceptibility testing (DST) by MGIT. For this assay, we included laboratory strain H37Rv (Rv), 7 isoniazid- and rifampicin-susceptible isolates (S1 to S7) (Fig. 3A), 19 isoniazid-resistant but rifampicin-susceptible isolates (IR1 to IR19) (Fig. 3B), and an MDR-TB isolate (R) (Fig. 3A and B), resistant to both isoniazid and rifampicin. For the MDK assay in clinical isolates, we used a higher rifampicin concentration of 2 μg/ml for both H37Rv and clinical M. tuberculosis isolates cultured without bead beating, and survival fraction was determined by the MPN method using a single set of serial dilution at different time points (Fig. 3A to H). The increase in antibiotic concentration increased the duration of maintaining the rifampicin concentration above the clinical susceptibility cutoff of 1 μg/ml in the medium. This extended the duration of the killing phase as well as the range of MDK for determining the rifampicin susceptibility among clinical M. tuberculosis isolates. Survival curves of the laboratory strain H37Rv and the MDR-TB isolate were included, with susceptible and isoniazid-resistant M. tuberculosis isolates, as controls for high rifampicin susceptibility and resistance, respectively (Fig. 3A and B).

All the rifampicin-susceptible M. tuberculosis isolates and H37Rv showed a reduction in viability, i.e., killing phase for 5 to 10 days of treatment compared with MPN/ml at day 0, whereas rifampicin-resistant MDR-TB isolates showed growth or maintenance of MPN/ml compared with day 0 values over 10 days of rifampicin treatment (Fig. 3A and B). From 5 to 10 days of rifampicin treatment, the regrowth phase was observed in most of the clinical M. tuberculosis isolates, potentially representing the outgrowth of *de novo* rifampicin-resistant cells (15). Since MDK time can be measured only during the killing phase of rifampicin treatment (29), the range of MDK measurements in our assay was restricted from 2 to 10 days of rifampicin treatment for isoniazid-susceptible (Fig. 3A) and -resistant isolates (Fig. 3B).

Although all the cultures were initially diluted to an optical density (OD) of 0.4, significant variation was observed in the initial MPN among clinical isolates. We observed that H37Rv showed the highest initial MPN, whereas clinical isolate initial MPN showed variation within the range of 10^4^ fold (Fig. 3A and B). This variation in the initial MPN was probably due to variation in cell size heterogeneity, cell envelope modifications, and clumping among clinical M. tuberculosis isolates. Even though initial MPN among clinical M. tuberculosis isolates showed variation, we could determine the MDK time for each of the isolates based on the time required for 2- and 4-log_10_ fold reductions in the MPN compared to its respective initial MPN (Fig. 3C to H).

There was substantial variation in population and subpopulation levels of rifampicin tolerance among susceptible and isoniazid-resistant M. tuberculosis isolates, as determined by approximate MDK_99_ and MDK_99.99_ times (Fig. 3C to H). To further confirm the robustness and reliability of the MDK assay for determining rifampicin tolerance of clinical isolates, we repeated the assay with 3 to 6 independent biological replicates of 6 strains, representing fully drug-susceptible (S7) and isoniazid-resistant (IR9, IR11, IR12, and IR13) strains and H37Rv (Fig. 3I and Fig. S3). The repeat assay fully recapitulated the observations, again revealing that 3/4 of the isoniazid-resistant isolates had longer MDK times for rifampicin than the fully drug-susceptible isolate (Fig. 3I and Fig. S3). Our data confirm the ability of the MDK assay to discriminate differences in the bulk population and subpopulation susceptibility and tolerance to rifampicin among clinical M. tuberculosis isolates.

**FIG 4:**
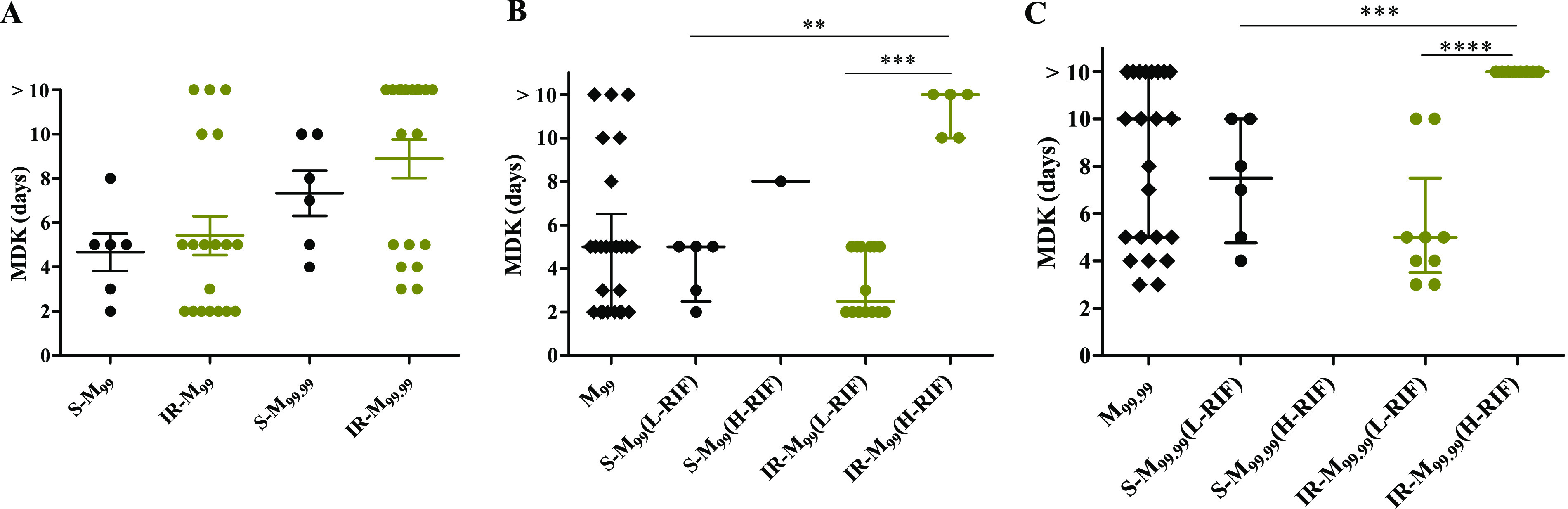
Variation in rifampicin tolerance as determined by MDK time. (A) MDK_99_ (M_99_) and MDK_99.99_ (M_99.99_) times for susceptible (S) and isoniazid-resistant (IR) isolates. (B and C) M_99_ (B) and M_99.99_ (C) for all susceptible and isoniazid-resistant isolates combined to determine the median (50th percentile) in the first column. Median values for M_99_ of 5 days and M_99.99_ of 10 days were used to group susceptible and isoniazid-resistant isolates with low (L-RIF < median) and high (H-RIF > median) rifampicin tolerance. Each data point represents one clinical M. tuberculosis isolate, and median and interquartile ranges of distributions are given (**, *P* < 0.01; ***, *P* < 0.001; ****, *P* < 0.0001; all by Mann-Whitney U test).

Finally, we investigated the variation in the distribution of rifampicin MDK_99_ and MDK_99.99_ times among isoniazid-susceptible and -resistant isolates. H37Rv showed high rifampicin susceptibility, similar to the earlier observation with 1-μg/ml rifampicin treatment, having an MDK_99_ and MDK_99.99_ time of less than 2 and 3 days, respectively, whereas for susceptible and isoniazid-resistant clinical M. tuberculosis isolates, MDK_99_ time distribution varied from 2 to >10 days and MDK_99.99_ time varied from 3 to >10 days; a value of more than 10 days indicates a high level of rifampicin tolerance beyond the detection limit of our assay (Fig. 4). There was no statistically significant difference between overall rifampicin tolerance distribution, measured as MDK_99_ and MDK_99.99_ time, between susceptible and isoniazid-resistant groups (Mann-Whitney U test) (Fig. 4A). We used the median of MDK_99_ (5 days) and MDK_99.99_ (10 days) distribution to group susceptible and isoniazid-resistant isolates with low (equal to or below the median) and high (above the median) rifampicin tolerance (Fig. 4A and B). All high-rifampicin-tolerance isolates were beyond the 75th percentile or above the upper detection limit of our assay (Fig. 4A and B). Out of 19 isoniazid-resistant isolates, 5 and 10 isolates showed significantly high rifampicin tolerance, as determined by MDK_99_ and MDK_99.99_, respectively, compared to susceptible and other isoniazid-resistant isolates (Fig. 4A and B). This further indicates the ability of the MDK assay to differentiate low and high rifampicin tolerance among clinical M. tuberculosis isolates.

## DISCUSSION

In this study, we developed an MPN-based MDK assay for determining the spectrum of rifampicin susceptibility in clinical M. tuberculosis isolates. Our MDK assay can be easily adapted to study the variation in killing dynamics and determine the susceptibility level for different conditions, such as different concentrations of rifampicin, other antituberculosis antibiotics, combinations of antibiotics, and host stresses or bacterial metabolic adaptations (such as different carbon sources) by changing the treatments accordingly. Our pilot-scale study indicates the presence of bulk population and subpopulation level variation in rifampicin susceptibility among clinical M. tuberculosis isolates, as determined by MDK_99_ and MDK_99.99_ times, respectively.

rifampicin-susceptible clinical M. tuberculosis isolates (fully susceptible and isoniazid resistant, *n* = 26) show variation in MDK time, ranging from less than 2 up to 10 days and greater than 10 days, which is beyond the detection limit of our assay using rifampicin at 2 μg/ml. This indicates the MDK assay can distinguish a spectrum of rifampicin susceptibility, ranging from highly susceptible to highly tolerant.

Tolerance or susceptibility assays require reliable methods to determine the survival fractions of bacterial populations at different durations after antibiotic exposure. Previous studies have shown that the MPN method quantitatively works as good as, and sometimes even better than, the CFU method for determining the viability of M. tuberculosis (24, 25). In addition, it has the advantage of incubating all serial dilutions, which is essential for determining the antibiotic tolerance among clinical M. tuberculosis isolates with wide variations in the spectrum of tolerance. Adopting the MPN method of the MDK assay in 96-well microtiter plates significantly reduces the labor required for testing antibiotic tolerance in large numbers of clinical isolates compared with the CFU method. Furthermore, the MPN method also reduces the time required for reading the results as the presence or absence of growth at each dilution (visual check) compared with the time required for counting colonies in the CFU method.

A striking observation of our study was the relatively increased tolerance to rifampicin in isoniazid-resistant isolates, particularly concerning MDK_99.99_. Routine laboratory MIC testing can miss such tolerance in clinical isolates (27). Importantly, the association of high rifampicin tolerance with isoniazid resistance may further contribute to the *de novo* emergence of multidrug resistance or failure of multidrug combinational therapy (17, 30) and requires further detailed investigation. The spectrum and high level of antibiotic tolerance observed in our clinical isolates is in accordance with the *in vitro* evolution of antibiotic tolerance in laboratory strains of mycobacteria (11) and high levels of antibiotic tolerance observed in other bacteria (31).

This assay has been developed considering laboratories based in low- and middle-income countries, and the growth on the microtiter plate can be read using a simple mirror box. The viability can be detected early using fluorescence dyes to further reduce the incubation time and obtain rapid results for clinical application (32). The MDK assay will also help us to screen for phenotypic antibiotic tolerance among clinical M. tuberculosis isolates and identify novel genetic variants and molecular mechanisms associated with the emergence of antibiotic tolerance (10–12). In addition, the MDK assay can help to identify possible chemical agents to reverse such tolerance by increasing the antibiotics potential (16). Investigating antibiotic tolerance and its emergence in clinical M. tuberculosis isolates will greatly improve treatment strategies by identifying hard-to-treat phenotypes and stratifying treatment approaches (33), preventing relapse or emergence of antibiotic resistance (17, 30).

## MATERIALS AND METHODS

### Bacterial isolates.

M. tuberculosis H37Rv and M. bovis BCG from a laboratory strain collection and clinical M. tuberculosis isolates from pulmonary tuberculosis patients, collected before and after treatment for a previous study (34), were revived from the archive and initially cultured in 7H9G medium (supplemented with 10% oleic acid-albumin-dextrose-catalase [OADC], 0.2% glycerol), followed by one or two subcultures in Middlebrook 7H9T medium (BD Difco, Thermo Fisher Scientific) supplemented with OADC and 0.05% Tween 80, with or without 0.2% glycerol. These cultures were used for the MDK experiments.

### Mycobacterium culture for MDK assay.

Mycobacterial isolates were cultured in 10 to 15 ml 7H9T medium in 50-ml tubes, with or without glass beads, in a shaking incubator at 37°C. When the culture reached an OD at 600 nm (OD_600_) range of 0.4 to 0.6, acid-fast staining was done to confirm the purity of the *Mycobacterium* culture. Cultures set with glass beads were vortexed for 3 min to disrupt bacterial aggregates, as the aggregates may influence the level of antibiotic tolerance (35). Cultures in both sets of tubes, with or without glass beads, were diluted to an OD_600_ of 0.4 with fresh 7H9T medium and used for the MDK assay.

### rifampicin preparation.

rifampicin (Sigma-Aldrich) stock solutions were prepared in dimethyl sulfoxide and filter sterilized, and aliquots were stored at −20°C for MDK assay.

### Bacterial viability determination by MPN method.

For the MDK assay, first the initial viability of mid-log-phase (OD_600_ of 0.4) M. tuberculosis cultures were measured by the MPN method (0 day) by taking a 1-ml aliquot from the culture at an OD of 0.4 and harvesting the culture at 7,000 rpm for 5 min. Nine hundred microliters of supernatant was removed carefully without disturbing the bacterial cell pellet, and the cell pellet was washed once by suspending in 1 ml (1× volume) of fresh 7H9T medium, vortexed, and recentrifuged. Nine hundred microliters of supernatant was removed, and the cell pellet again was resuspended in 1 ml fresh 7H9T medium. One hundred microliters from this 1-ml culture was transferred to 96-well plates as an undiluted culture in triplicate for serial dilution. Ten microliters from the undiluted (10^0^) culture was used for serial dilution in 90 μl 7H9T medium up to 10^−9^ dilutions in microtiter plates (Fig. 1). Each time, new pipette tips were used for mixing and transferring culture during serial dilution. The final two wells, with 7H9T medium at the end of serial dilution after 10^−9^, were left for sterility check without adding any mycobacterial culture (Fig. 1). The serially diluted plates were sealed by 96-well plastic adhesive seal and incubated at 37°C, without shaking, for 1 or 2 months. Immediately after initial (0 day) viability measurement, the mycobacterial cultures in the 50-ml tubes were treated with rifampicin at a final concentration of 1 or 2 μg/ml, and cultures were further incubated at 37°C with shaking (Fig. 1). At different time points (1, 2, 3, 5, 10, 15, and 20 days, depending on the experiment) after rifampicin treatment, viable bacterial numbers were measured again by removing 1 ml from the rifampicin-treated culture, cells were washed once with 1 ml fresh 7H9T medium (1× volume) to remove rifampicin, and the MPN assay was repeated as performed on the initial day (0 day) before rifampicin treatment (Fig. 1). In addition, we performed the rifampicin MDK assay and viability measurement using the CFU method for H37Rv. For CFU-based viability determination, the same steps were followed as those described for the MPN method, except 50-μl aliquots from different serial dilutions were plated on 7H10 plates and incubated at 37°C, and CFU numbers were counted after 30 days of incubation.

For the MPN method, growth in 7H9T medium at the bottom of 96-well plates was recorded by the Thermo Fisher Sensititre Vizion digital MIC viewing system (Thermo Fisher Scientific, Inc.) after 15 to 20 days and 1 and 2 months of incubation (Fig. 1; see also Fig. S1 in the supplemental material). Final MPN results were calculated based on 15 to 20 days of incubation for antibiotic tolerance determination between clinical M. tuberculosis isolates considering different factors influencing the MPN (Fig. S1). There was some increase in MPN at 1 month (slow growth possibly due to postantibiotic effect or resuscitation of nonreplicating persisters), and drying up of wells was also observed in 96-well plates at the 1- and 2-month time points (Fig. S1). Early reading at 15 to 20 days clearly distinguished differences in the extent of growth even at the same serial dilution between M. tuberculosis isolates (Fig. S1A). Due to higher sensitivity in detecting differences in growth at 15 to 20 days of incubation and drying up of wells at later incubation periods, we used 15- to 20-day MPN results for calculating the survival fraction after rifampicin treatment (Fig. S1). M. tuberculosis viability at each time point is calculated as mean MPN/ml with 95% CI (confidence intervals) or with standard deviations. For a single set of dilution series, the MPN/ml was determined by the highest dilution showing growth multiplied by the dilution factor. Based on MPN value at each time point, survival curves were plotted to determine the killing dynamics after rifampicin treatment. The MDK_99_ and MDK_99.99_ times were calculated based on the duration required for 99% and 99.99% reduction in the survival of the population, respectively, during the killing phase compared to the initial MPN/ml, taken as 100%.

### DST.

MGIT was used for phenotypic drug susceptibility testing (DST) by a Bactec MGIT 960 SIRE kit (Becton, Dickinson) according to the manufacturer’s protocol. Bactec MGIT 960 SIRE DST was done for streptomycin (1.0 μg/ml), isoniazid (0.1 μg/ml), rifampicin (1.0 μg/ml), and ethambutol (5.0 μg/ml) (34).

### GeneXpert MTB/RIF.

For the GeneXpert MTB/RIF detection of rifampicin resistance, we followed the manufacturer’s protocol using the GeneXpert instrument, GeneXpert Dx system software, and cartridge (Cepheid) (36). Briefly, 0.2 ml of decontaminated sputum was added into 2 ml of sample reagent and transferred into a test cartridge. The cartridge was inserted into the test platform of a GeneXpert instrument and postrun analyzed for detection of M. tuberculosis and rifampicin resistance (36).

## Supplementary Material

Supplemental file 1
