# Supplementary material for: Most-Probable-Number-Based Minimum Duration of Killing Assay for Determining the Spectrum of Rifampicin Susceptibility in Clinical Mycobacterium tuberculosis Isolates
Source: Antimicrob Agents Chemother. 2021 Feb 17;65(3):e01439-20. doi: 10.1128/AAC.01439-20 (PMC8092508; doi:10.1128/AAC.01439-20)
Supplement: Supplemental file 1 [file AAC.01439-20-s0001.pdf]

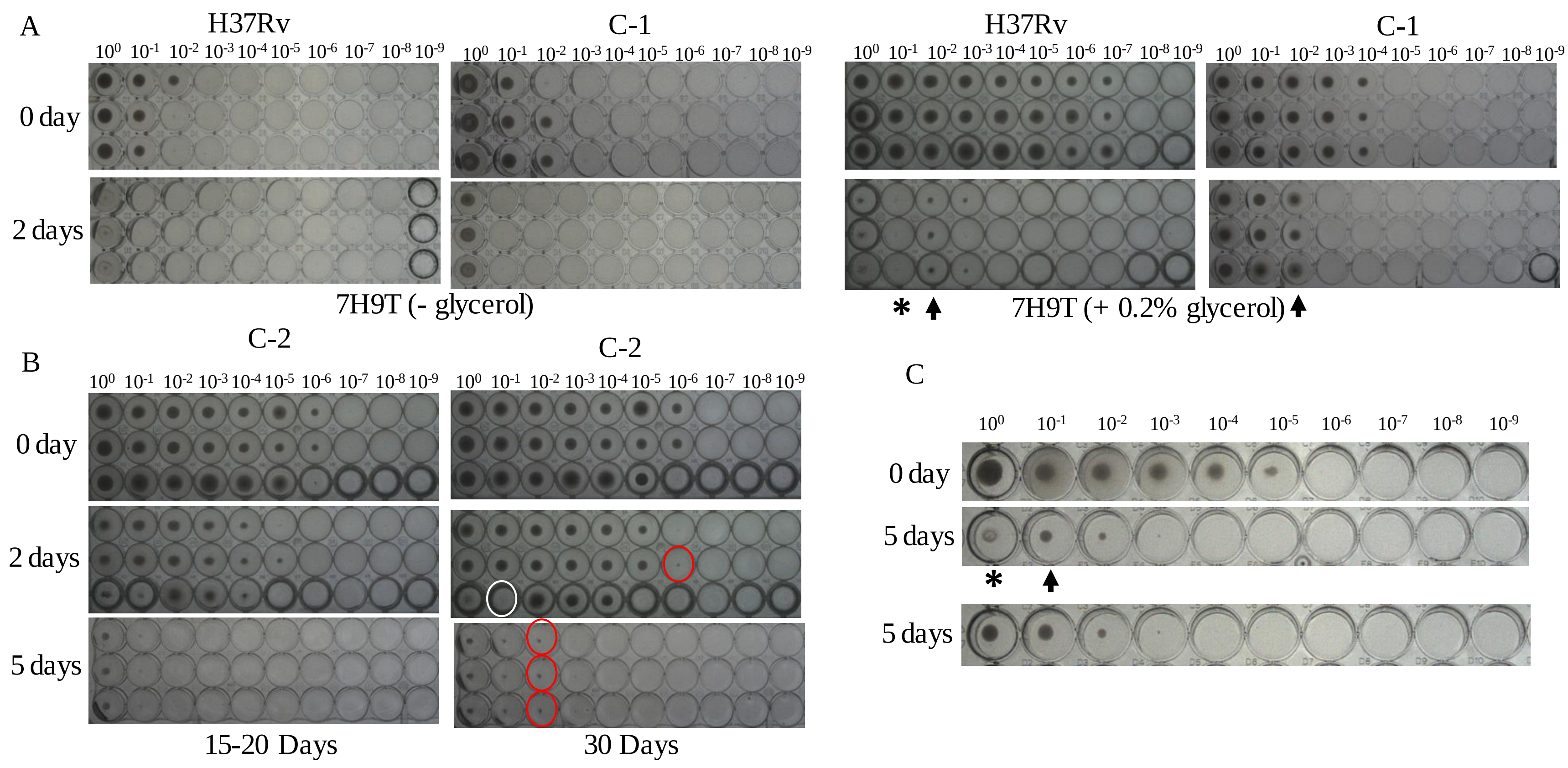

Supplementary Figure 1. Factors influencing MPN number and survival fraction of *M. tuberculosis* isolates in rifampicin MDK assay. (A) Mid-log cultures of laboratory strain H37Rv, and clinical *M. tuberculosis* isolate (C-1) treated with 2 µg/mL of rifampicin. MPN assay plates showing the difference in MPN number due to presence or absence of glycerol in 7H9T medium at 0 and post 2 days of rifampicin treatment. We observed consistently higher MPN/mL in the presence of glycerol, whereas MPN/mL varied as high or low between experiments in cultures without glycerol. *Indicates low or undetectable growth in lower serial dilution as compared to next higher dilutions with clear growth, in such cases the highest dilution showing growth was considered for MPN calculation (as longer incubation resulted in clear growth in lower dilution also). Arrow head compares difference in the extent of growth in same serial dilution between H37Rv and C-1 at 15-20 days of incubation. This shows that even though MPN number may be same, difference in growth further indicate difference in survival fraction and rifampicin tolerance between H37Rv and C-1. (B) Clinical *M. tuberculosis* isolate (C-2) rifampicin MDK assay (0, 2 and 5 days post 2 µg/mL of rifampicin treatment) vizion images of same microtiter plate taken at 15-20 days and 30 days incubation time. White circle shows dried well at 30 days of incubation, red circle show increase in MPN at 30 days compared to 15-20 days of incubation. (C) Residual rifampicin in *M. tuberculosis* culture aliquots, post-rifampicin treatment and washing, can inhibit or reduce the growth in undiluted or lower dilution wells (* - indicate reduced cell growth resulting in slightly light/gray colored spot) as compared to subsequent higher dilution showing dark bacterial growth spot, indicated by black arrow in middle 5 days MPN dilution series. Bottom most 5 days dilution series shows example for proper washing and growth with similar dark colored growth in all serial dilutions.

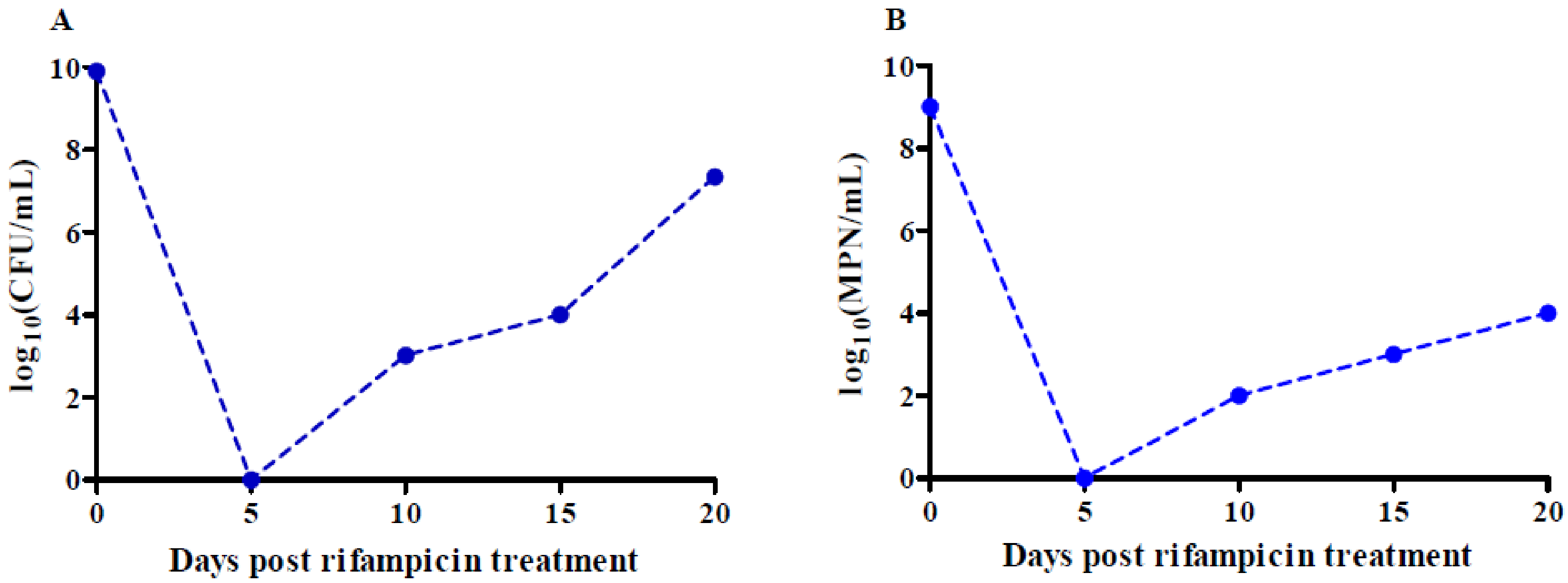

Supplementary Figure 2. H37Rv isolate survival and re-growth post rifampicin treatment. (A) Rifampicin MDK assay for mid-log cultures of H37Rv treated with 5 µg/mL of rifampicin and viability determined by CFU method for 20 days post-rifampicin treatment**.** (B) Rifampicin MDK assay for mid-log cultures of H37Rv, treated with 2 µg/mL of rifampicin and viability determined by MPN method for 20 days post-rifampicin treatment.

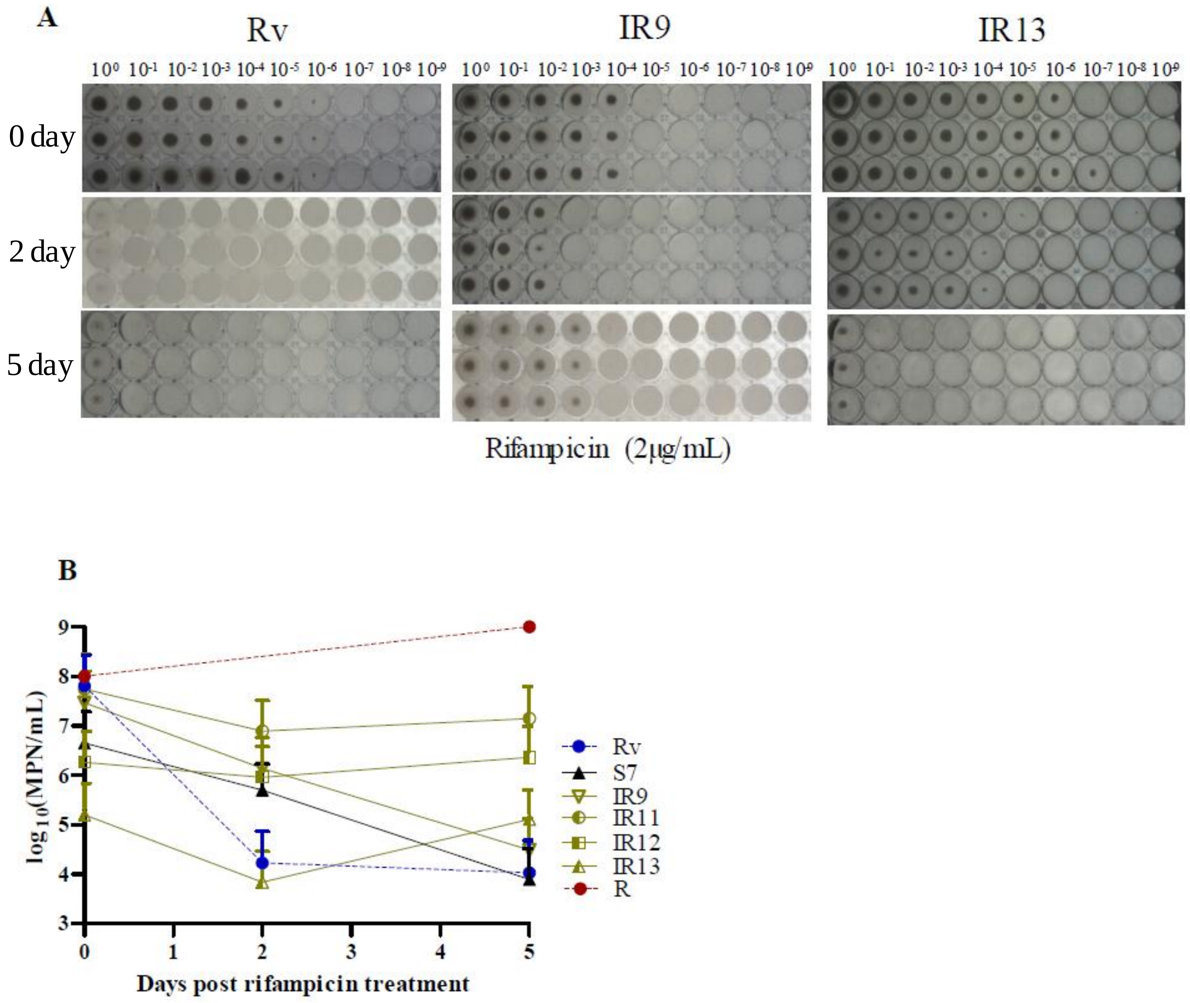

Supplementary Figure 3. Robustness of MDK assay in determining the spectrum of rifampicin susceptibility in *M. tuberculosis* isolates. Mid-log cultures of laboratory strain H37Rv (Rv), isoniazid and rifampicin susceptible isolate (S7), isoniazid resistant clinical *M. tuberculosis* isolates (IR9, IR11, IR12 and IR13), were vortexed with beads and treated with 2 µg/mL of rifampicin (3 to 6 biologically independent experiments, except MDR-TB (R) only had result from single experiment). (A) MPN assay plates showing the spectrum of rifampicin susceptibility between H37Rv, IR9 and IR13 for 0, 2 and 5 days of rifampicin treatment after 15-20 days of incubation. (B) Average viable mycobacterial cell number as determined by MPN method at 0 day (just before rifampicin treatment), 2 and 5 days post rifampicin treatment.
